# High- and Low-Energy Lisfranc Injuries: A Prospective Study of Functional Outcomes and an Algorithm Based on Trauma Kinetics

**DOI:** 10.7759/cureus.100618

**Published:** 2026-01-02

**Authors:** Daniel F Kafury, Jorge Hernandez, Carlos E Ramirez, Juan B Gerstner

**Affiliations:** 1 School of Medicine, Universidad Icesi, Cali, COL; 2 Department of Orthopaedic Surgery, Universidad del Valle, Cali, COL; 3 Department of Foot and Ankle Surgery, Universidad del Valle, Cali, COL

**Keywords:** algorithm-based treatment, functional and clinical outcome, internal fixation, lisfranc joint injury, midfoot fracture-dislocation

## Abstract

Introduction

Lisfranc joint injuries remain a diagnostic and therapeutic challenge due to their low incidence, variable presentation, and potential for long-term functional impairment. This study aimed to evaluate functional outcomes in patients with high- and low-energy Lisfranc fracture-dislocations using a standardized treatment algorithm based on trauma kinetics and chronicity.

Methods

We conducted a prospective descriptive case series including 32 adult patients treated at a tertiary referral center in southwestern Colombia between January 2021 and June 2023. Injuries were classified using the Myerson system for high-energy trauma and the Nunley-Vertullo system for low-energy ligamentous injuries. A clinical decision-making algorithm was applied to guide surgical or conservative management. All patients were followed for a minimum of 12 months, and outcomes were assessed using the Visual Analog Scale (VAS), the American Orthopaedic Foot and Ankle Society (AOFAS) Midfoot Score, return-to-work status, and need for footwear modification.

Results

Of 32 patients, 21 sustained high-energy injuries (all treated surgically), and 11 sustained low-energy injuries (10 treated conservatively). At six months, mean VAS scores decreased from 8 to 2 in the high-energy group and from 6 to 3 in the low-energy group. Mean AOFAS scores were 78 and 81, respectively. Return-to-work rates were 100% and 73%, respectively. Permanent footwear adaptation was required in 9% of cases.

Conclusion

A treatment algorithm based on injury kinetics and chronicity offers a reproducible and structured approach to the management of Lisfranc injuries. The favorable outcomes observed in this series support its clinical utility, particularly in high-volume trauma settings.

## Introduction

Lisfranc joint injuries are relatively rare and exhibit variable incidence. They are estimated to affect approximately 1 in 60,000 individuals annually and account for 0.2%-0.9% of all skeletal fractures, although their true prevalence may be underestimated due to diagnostic challenges in early stages [1-3]. These injuries can result from either indirect or direct mechanisms, with indirect trauma being the most common. Indirect mechanisms typically involve axial loading of the plantar-flexed forefoot or forced abduction of the midfoot [4-6]. In contrast, direct trauma, such as high-energy impacts or crush injuries, is less frequent but is often associated with worse outcomes and a higher risk of complications, including compartment syndrome [6,7].

In various case series, Lisfranc joint injuries have shown diagnostic delays in more than 20% of patients, particularly in low-energy trauma, which is associated with adverse outcomes such as chronic pain syndrome and midfoot collapse [4,7].

High-energy Lisfranc injuries are the most frequently diagnosed. These lesions are typically classified according to the Quénu and Küss system, as modified by Hardcastle et al. [6], and later refined by Myerson et al. [7] and Myerson et al. [8]. This classification divides injuries into three categories: type A (total incongruity), type B (partial incongruity; B1: medial, B2: lateral), and type C (divergent) [9,10].

For low-grade injuries, the classification proposed by Nunley and Vertullo is recommended. This system defines three grades: grade I (ligamentous sprain), grade II (ligament rupture with 2-5 mm diastasis between the first and second metatarsals without collapse of the medial longitudinal arch), and grade III (rupture with associated arch collapse) [4].

The most commonly described clinical manifestations include midfoot pain, swelling, and ecchymosis. For diagnostic purposes, initial imaging should include anteroposterior, lateral, and 30-degree oblique radiographs of the foot. If no significant abnormalities are observed, stress radiographs may be considered. The primary goal of radiographic assessment is to identify fractures or indirect signs of Lisfranc ligament complex injury, such as misalignment of the second tarsometatarsal joint, lateral displacement of the second metatarsal relative to the second cuneiform, or a diastasis greater than 2 mm between the first and second tarsometatarsal joints. However, in the acute trauma setting, stress radiographs are often poorly tolerated, making computed tomography (CT) a fundamental diagnostic tool.

In selected cases where indirect radiographic signs are absent but clinical suspicion remains high, magnetic resonance imaging (MRI) can be useful to identify Lisfranc ligament disruption, ligamentous elongation, or periligamentous edema [2,11,12].

The main goal of treatment is to achieve anatomical reduction and stable fixation to restore a plantigrade, pain-free, and functional foot, as this has the greatest impact on long-term clinical outcomes [4,7,13]. Several variables have been described to guide treatment decisions, including trauma mechanism, radiographic findings, and the patient’s pre-injury activity level. While surgical management is generally preferred, conservative treatment may be considered in cases of pure ligamentous injuries or low-grade trauma with preserved joint stability [14,15].

Timely diagnosis and appropriate treatment decisions are critical, as untreated or inadequately managed Lisfranc injuries may result in significant complications, including post-traumatic osteoarthritis in up to 25% of cases, persistent pain, joint stiffness, complex regional pain syndrome, gait disturbances, and collapse of the plantar arch [4,7,16-19].

Despite multiple classification systems, a validated decision-making protocol integrating trauma kinetics and chronicity is lacking in the literature. The objective of this study is to describe the management of Lisfranc fracture-dislocations at a tertiary referral hospital in southwestern Colombia between January 2021 and June 2023. This includes differentiation between high- and low-energy trauma to propose a standardized management algorithm based on injury kinetics and timing, as well as analysis of functional outcomes with a minimum one-year follow-up.

This article was previously presented as an oral presentation at the 70th Colombian Society of Orthopedic Surgery and Traumatology (SCCOT) National Congress, held from May 28 to 31, 2025, in Cartagena, Colombia.

## Materials and methods

A prospective descriptive observational case series was conducted at Hospital Universitario del Valle, a tertiary referral center in southwestern Colombia, between January 2021 and June 2023. A total of 32 adult patients presenting with Lisfranc joint injuries were consecutively enrolled and treated according to a clinical algorithm developed by the research team. The algorithm incorporates trauma energy (high versus low), injury chronicity (acute versus delayed), and radiographic instability, and was designed based on a narrative review of the literature and the clinical experience of a nationally and internationally recognized foot and ankle subspecialist.

Study population

Inclusion criteria were adults (aged ≥18 years) with acute or subacute Lisfranc joint injuries confirmed through clinical and radiographic evaluation. Lesions included pure dislocations, fractures, and fracture-dislocations. Exclusion criteria were prior injury or surgery on the affected foot, degenerative or rheumatologic midfoot conditions, diabetic neuropathy, peripheral vascular disease, or history of primary or secondary arthrodesis.

Management algorithm and data collection

After initial assessment, injuries were classified using the Myerson system for high-energy trauma and the Nunley and Vertullo classification for low-energy ligamentous injuries. Treatment modality (surgical or conservative) was determined based on the algorithm. Conservative treatment involved a below-knee plaster cast for two weeks with strict non-weight-bearing, followed by transition to a walker boot and progressive weight-bearing as tolerated based on clinical and radiological findings.

Surgical patients underwent open reduction and internal fixation (ORIF) with 2.7 mm low-profile plates and screws, after temporary stabilization using Kirschner wires, or the TightRope® suture button system.

Demographic data, injury mechanism, classification, associated injuries, treatment type, complications, and follow-up outcomes were recorded. All patients completed at least 12 months of follow-up.

Outcome measures

Pain was measured using the Visual Analog Scale (VAS), and function was assessed using the American Orthopaedic Foot and Ankle Society (AOFAS) Midfoot Score. Patient satisfaction (0-10 scale), return to work status, and footwear modifications were also documented.

Statistical analysis

Data were anonymized and entered into Microsoft Excel version 16.16.11 (Microsoft Corp., Redmond, WA). Descriptive statistics were used to report demographic and clinical characteristics. Given the case series design and limited sample size, no inferential statistical tests were applied.

## Results

During the two-and-a-half-year period from January 2021 to December 2023, a total of 32 patients with Lisfranc fracture-dislocations were identified. Of these, 31 were initially assessed in the emergency department, while one case was diagnosed two weeks after the trauma during an outpatient consultation, having been missed at the initial evaluation.

Of the total sample, 66% (n = 21) sustained high-energy trauma, and 34% (n = 11) sustained low-energy trauma. The mean age was 30 years in the high-energy group and 43 years in the low-energy group. Regarding sex distribution, 81% (n = 19) of the high-energy cases were male, compared to 63% (n = 7) in the low-energy group (Table 1).

**Table 1:** Baseline sociodemographic and clinical characteristics of patients with Lisfranc injuries Data are presented as number (%) for categorical variables and mean ± SD for continuous variables. No inferential statistical tests were applied, given the descriptive nature of the study. Statistical significance, when applicable, was defined as p < 0.05. SD: standard deviation, VAS: Visual Analog Scale

Variable	High-energy (n = 21)	Low-energy (n = 11)	Total (N = 32)
Mean age (years), range	30 (18-45)	43 (32-68)	38 (18-68)
Male sex, number (%)	19 (90%)	7 (63%)	26 (81%)
Right-sided injury, number (%)	62%	58%	60%
Initial VAS score, mean (SD)	8.0	6.0	7.0
Myerson classification, number (%)	21 (100%)	-	21 (66%)
• Type A	8	-	8
• Type B1/B2	5/6	-	5/6
• Type C	2	-	2
Nunley-Vertullo classification, number (%)	-	11 (100%)	11 (34%)
• Grade I	-	7	7
• Grade II	-	3	3
• Grade III	-	1	1
Delayed diagnosis, number (%)	0	1 (9%)	1 (3%)
Associated injuries (only high-energy)	-	-	-
• Traumatic brain injury, number (%)	8 (38%)	0	8 (38%)
• Thoracic trauma, number (%)	5 (23%)	0	5 (23%)
• Abdominal trauma, number (%)	3 (14%)	0	3 (14%)
• Facial injuries, number (%)	2 (9%)	0	2 (9%)
• Arterial vascular injury, number (%)	1 (5%)	0	1 (5%)

All patients with high-energy trauma underwent surgical treatment. In the low-energy group, only one patient required surgery, having been classified as Nunley-Vertullo grade III; the remaining cases were managed conservatively.

Initial pain scores using the Visual Analog Scale (VAS) were 8 in the high-energy group and decreased to 2 at six-month follow-up. In the low-energy group, the mean VAS score decreased from 6 to 3 over the same period. Functional outcomes assessed by the American Orthopaedic Foot and Ankle Society (AOFAS) Midfoot Score averaged 78/100 in the high-energy group and 81/100 in the low-energy group at six months (Table 2).

**Table 2:** Functional outcomes at six-month follow-up Data are presented as mean ± SD or number (%), as appropriate. No inferential statistical tests were applied, given the descriptive design and small sample size. When applicable, statistical significance was defined as p < 0.05. AOFAS: American Orthopaedic Foot and Ankle Society, SD: standard deviation, VAS: Visual Analog Scale

Outcome variable	High-energy (n = 21)	Low-energy (n = 11)	Total (N = 32)
VAS at admission, mean (SD)	8.0	6.0	7.0
VAS at six months, mean (SD)	2.0	3.0	3.5
AOFAS midfoot score, mean (SD)	78/100	81/100	80/100
Return to work at six months, number (%)	21 (100%)	8 (73%)	29 (91%)
Permanent footwear adaptation, number (%)	2 (10%)	1 (9%)	3 (9%)
Patient satisfaction (0-10), mean (SD)	9/10	8/10	8/10
Time to weight-bearing (weeks), mean	3	4	3
Reintervention required, number (%)	2 (9.5%)	0	2 (6.2%)

Footwear modification was required in two patients from the high-energy group (2/19) at six months, while one patient in the low-energy group-who had been diagnosed late-required permanent shoe adaptation.

Regarding return to work, all 21 patients in the high-energy group resumed their pre-injury occupational activities by six months. In contrast, three patients in the low-energy group (3/11) had not returned to work at that time.

Associated injuries in high-energy trauma cases included traumatic brain injury in 38% (n = 8), thoracic trauma in 23% (n = 5), abdominal trauma in 14% (n = 3), facial injuries in 9% (n = 2), and one case of arterial vascular injury. No cases of compartment syndrome or fasciotomy were reported.

## Discussion

Tarsometatarsal joint injuries (Lisfranc injuries) are rare but should be regarded as highly disabling lesions due to their anatomical location and the biomechanical demands of the midfoot during standing and gait. Delayed diagnosis, reported in up to 20% of cases in the literature [3,19], is associated with poorer functional outcomes. This is particularly relevant considering that young adults are the most frequently affected population. In our series, the rate of delayed diagnosis was notably low (3%; 1/32), likely influenced by a prior targeted training program conducted in the emergency department to raise awareness of Lisfranc injuries.

A persistent challenge in managing these injuries is the choice of an appropriate classification system. Among high-energy injuries, the Myerson classification remains one of the most practical and widely adopted. In our cohort, 66% of cases were high-energy injuries, a proportion notably higher than typically reported [1,20,21]. This may be explained by the setting of the study in Cali, Colombia, a city with a high incidence of motor vehicle-related trauma.

The primary surgical goal is anatomical reduction, which, as described by Alcelik et al. [3], is a key factor in preventing post-traumatic osteoarthritis, improving postoperative AOFAS scores, and reducing the incidence of chronic pain. Based on our findings, we recommend open reduction and internal fixation using 2.7 mm low-profile plates in patients with high-energy trauma, regardless of Myerson subtype, and in low-energy injuries classified as Nunley-Vertullo grade III. We propose a standardized fixation sequence beginning with the first tarsometatarsal joint, followed by temporary fixation of the second and third metatarsals with Kirschner wires, and finally stabilization of the mobile column (fourth and fifth metatarsals). This method provides greater stability, facilitates earlier mobilization, and may reduce secondary displacement and the risk of secondary arthrosis.

According to our institutional experience, TightRope® endobutton systems may also be considered in selected cases, depending on fracture morphology and surgeon preference. Kirschner wires should be reserved for the lateral column, associated injuries, or compromised soft tissue conditions [2,22-24]. Ho et al. compared the stability of screw fixation versus plating and found no statistically significant differences between the two methods [5].

Although several recent publications support primary arthrodesis in acute Lisfranc injuries, current evidence remains inconclusive. Alcelik et al. conducted a meta-analysis comparing ORIF versus primary arthrodesis and concluded that functional outcomes were similar between groups, although ORIF was associated with a higher risk of implant removal [3].

We believe that the decision between these two strategies should be individualized based on patient-specific factors, including comorbidities, soft tissue status, trauma mechanism, and fracture pattern. The initial surgical approach should aim for cost-effectiveness, implant durability, and improved quality of life. However, based on our clinical protocol, we advocate for joint preservation in all acute cases and therefore do not recommend primary arthrodesis in this setting.

Functional outcomes in our series, achieved through the application of a standardized algorithm, were superior to those reported in the meta-analysis and systematic review by Alcelik et al. [3,23]. These findings support the potential of the proposed algorithm as a reproducible and replicable strategy with outcomes comparable or potentially superior to those documented in current literature.

Rates of return to work and permanent footwear adaptation in our cohort were also higher than those previously reported, reinforcing the importance of proper patient selection based on trauma kinetics and timing [3]. In selected cases, conservative treatment may yield comparable results to surgical management when guided by strict clinical criteria. These results diverge from some reports in the literature and suggest that tailored management based on classification and trauma characteristics may be equally effective [3,4,22,24].

**Figure 1:**
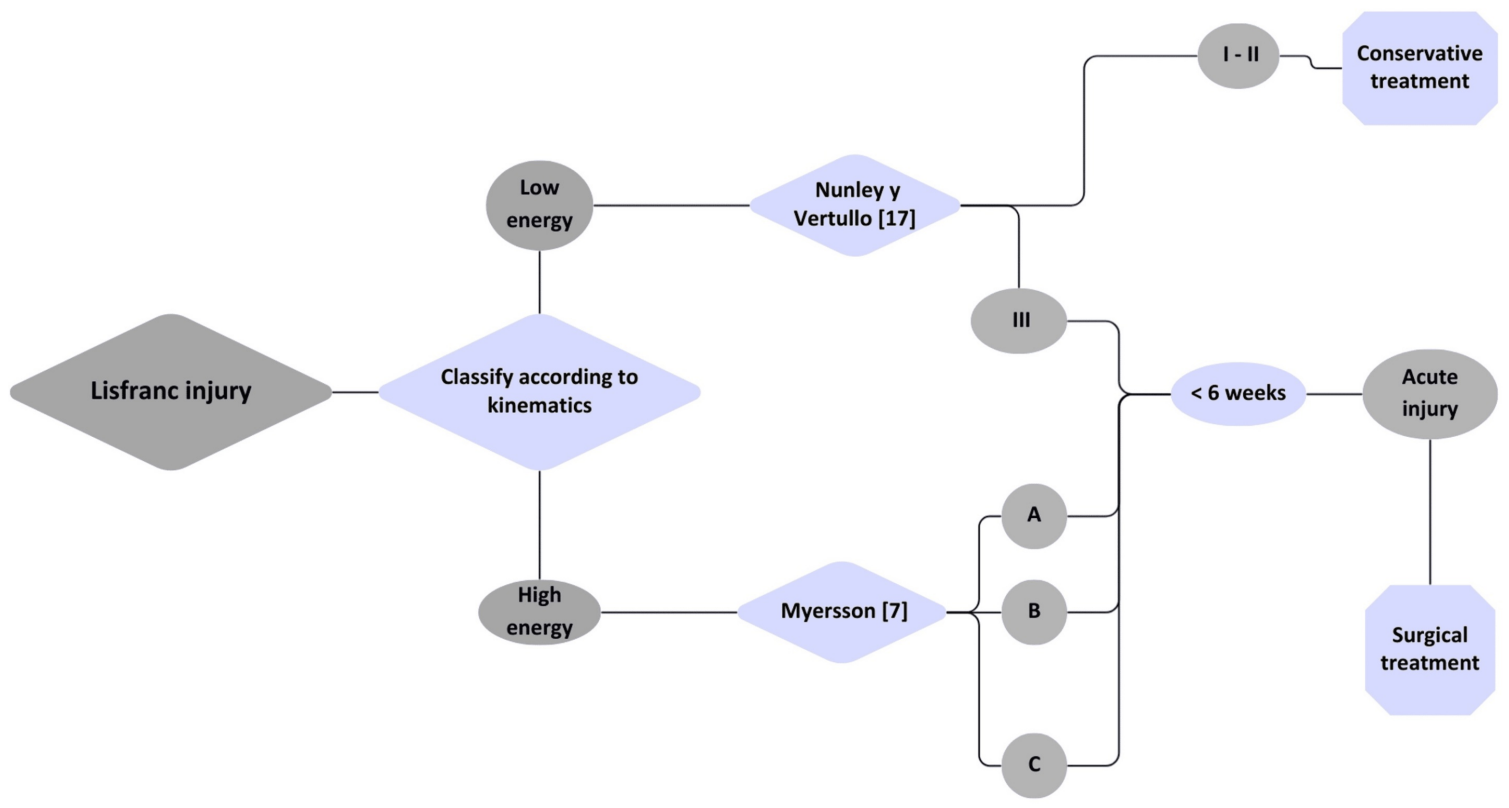
Treatment algorithm Sources: Myerson MS, Fisher RT, Burgess AR, Kenzora JE: Fracture dislocations of the tarsometatarsal joints: end results correlated with pathology and treatment. Foot Ankle. 1986, 6:225-42. 10.1177/107110078600600504 [7] and Nunley JA, Vertullo CJ: Classification, investigation, and management of midfoot sprains: Lisfranc injuries in the athlete. Am J Sports Med. 2002, 30:871-8. 10.1177/03635465020300061901 [17]

Based on the literature review and our case series, we propose a treatment algorithm designed to assist clinicians in managing Lisfranc joint injuries efficiently and systematically (Figure 1).

Strengths of the study

This study presents several strengths that enhance the relevance and clinical utility of its findings. First, it includes a prospective case series with consecutive patient enrollment over a well-defined time frame, ensuring methodological consistency and reducing selection bias.

Second, the implementation and evaluation of a structured management algorithm based on trauma kinetics and timing provide a novel contribution to the literature. To our knowledge, few studies have applied a standardized protocol to guide surgical versus conservative decision-making in Lisfranc injuries.

Third, all patients were followed for a minimum of 12 months, allowing for meaningful assessment of mid-term functional outcomes using validated instruments (VAS and AOFAS scores), as well as real-world endpoints such as return to work and footwear modification.

Finally, the study was conducted in a high-volume trauma center within a region of elevated incidence of high-energy injuries, offering insights applicable to similar urban or resource-limited settings, particularly in Latin America, where such data are scarce.

Clinical implications

The management of Lisfranc joint injuries remains highly variable across institutions, often influenced by physician experience and local protocols. The findings of this study suggest that a standardized, classification-driven algorithm based on trauma kinetics and injury chronicity may streamline clinical decision-making and improve consistency in treatment approaches.

By incorporating validated classification systems (Myerson and Nunley-Vertullo), along with time-based stratification (acute versus chronic presentation), the proposed algorithm allows for early identification of cases requiring surgical intervention and avoids overtreatment in stable, low-grade injuries. This approach not only optimizes resource allocation but also supports joint preservation in acute cases, aligning with long-term functional goals.

Furthermore, the algorithm’s application in a high-volume trauma center with favorable functional outcomes and high return-to-work rates reinforces its potential adaptability in similar clinical settings. Widespread adoption of such a structured model could enhance early diagnosis, reduce variability in care, and contribute to better long-term outcomes in patients with Lisfranc injuries.

Limitations

This study presents several limitations inherent to its design. First, the observational nature limits the generalizability of the findings and precludes robust statistical analysis to establish causal associations. Although patients were prospectively enrolled and evaluated using a standardized management algorithm, the absence of a control group managed without the algorithm hinders direct comparisons.

Second, the study was conducted at a single tertiary referral center in southwestern Colombia, which may introduce selection bias, particularly given the high incidence of high-energy trauma in this geographic region. This epidemiologic profile may not reflect patterns observed in other populations or healthcare settings.

Third, the functional outcome measures (VAS, AOFAS score, and return to work), while widely used and clinically meaningful, rely in part on subjective reporting. Additionally, longer-term complications such as post-traumatic arthritis may not be fully captured within the minimum one-year follow-up period.

Future studies with larger, multicenter cohorts, longer follow-up, and comparative designs (e.g., randomized controlled trials) are needed to validate the effectiveness and reproducibility of the proposed management algorithm.

## Conclusions

Lisfranc joint injuries, although uncommon, have a high potential for long-term functional impairment if not promptly diagnosed and appropriately treated. Our findings support the utility of a clinical algorithm based on trauma kinetics, injury chronicity, and validated classification systems (Myerson and Nunley-Vertullo) to guide the decision-making process between surgical and conservative management.

The application of this algorithm in a prospective case series demonstrated favorable functional outcomes, high rates of return to work, and low incidence of complications, even in a setting with a predominance of high-energy trauma. These results suggest that a standardized, individualized approach can improve clinical consistency and optimize long-term outcomes in patients with Lisfranc injuries. Further multicenter studies with larger cohorts and extended follow-up are warranted to validate these findings and assess the algorithm’s applicability across diverse healthcare settings. We believe the proposed algorithm may serve as a practical reference for clinicians managing Lisfranc injuries, particularly in resource-limited or high-trauma settings.
